# Association of Vitamin B12 deficiency with long-term PPIs use: A cohort study

**DOI:** 10.1016/j.amsu.2022.104762

**Published:** 2022-09-26

**Authors:** Hassan Mumtaz, Bushra Ghafoor, Hina Saghir, Mariam Tariq, Kashmala Dahar, Syed Hasan Ali, Syeda Tahira Waheed, Abdul Ahad Syed

**Affiliations:** a Clinical Research Associate, Maroof International Hospital, Islamabad, Pakistan; b Public Health Scholar, Health Services Academy, Islamabad, Pakistan; c DG Khan Medical College, Dera Ghazi Khan, Pakistan; d HBS Medical and Dental College, Pakistan; e Chandka Medical College Larkana, Pakistan; f Dow University of Health Sciences, Pakistan; g Liaquat National Medical College and Hospital, Pakistan

**Keywords:** Proton pump inhibitors, Vitamin B12 deficiency, PPI, Clostridium difficile-associated diarrhea

## Abstract

**Background:**

Proton Pump inhibitors are widely used among the majority of the world's population as acid-suppressing medications. Proton Pump Inhibitors have been reported to cause intestinal damage and adverse gut microbiota changes affecting several mechanisms, including malabsorption, etc.

**Aim:**

In order to gain a deeper understanding, we conducted a cohort analysis to assess the prevalence & association of Vitamin B12 deficiency in patients on long-term use of PPIs.

**Methods:**

This single-center cohort study was conducted at the Department of Internal Medicine, KRL hospital in Islamabad, Pakistan from May 2021 to May 2022. Rao soft calculator with a 95% confidence interval and 5% error margin was used to find the estimated sample size. Vitamin B_12_ levels were analyzed using the Cobas e411 analyzer. Chi-square test, odds ratio, and t-tests were used for analysis.

**Results:**

Among the 1225 participants, more than half of the men (55.10%) had low levels of vitamin B12. Vit B12 levels were observed to be significantly lower in Omeprazole patients than in Pantoprazole patients. A vitamin B12 deficiency is 0.5 times more likely in patients taking PPIs. There is a substantial difference between the early and final levels of B12 indicated by the *t*-test.

**Conclusion:**

According to our findings, long-term usage of PPIs is linked to an increased risk of vitamin B12 insufficiency specifically in men falling under the ages of 18 and 40.

## Highlights

• Increased focus and precision have been achieved by single-centered approach.
• Over 1200 people were enrolled during the course of the year-long data collection period, which have contributed to the study's overall authenticity.
• It is possible that this large-scale investigation may serve to clarify the link between PPIs and VIT B12 deficiency during this period of uncertainty.

## 1 Introduction

Proton Pump inhibitors are widely used among the majority of the world's population as acid-suppressing medications, and the majority of patients who receive PPIs do so for at least one year without a clear indication [1]. Among 700 different drugs, Proton Pump Inhibitors have also been reported to cause intestinal damage and adverse gut microbiota changes affecting several mechanisms, including malabsorption, autoimmune changes, and other microscopic complications [2].

In addition, patients who continue to take proton pump inhibitors (PPIs) for a long period experience decreased gastric acid secretion and lowered vitamin B-12 absorption [[3], [4], [5], [6]]. This statement is supported by some systematic reviews and meta-analyses [7,8] as well as a substantial number of population-based studies conducted in which data accounts for the increased risk of vitamin B12 deficiency in patients who take PPIs at rates of 1.5 PPI pills per day for more than 2 years [9]. Acid suppression therapy encompasses a wide range of drugs, including H2 receptor blockers as well as PPIs that cause Vitamin B12 deficiency, regardless of whether the agents are of the same class or different [10]. Considering the time duration, multiple studies demonstrate different results than those expected. PPIs such as Omeprazole, if used for more than 6 months can cause Vitb12 deficiency clinical manifestations including Neurotoxicity and cognitive decline by oxidative stress mechanisms [11].

However, when the duration of therapy was over 4 years and routine monitoring was not taken into account, Omeprazole and Pantoprazole did not contribute to Vitamin B12 deficiency [12]. A cross-sectional study that was conducted on patients with Peptic ulcer disease who had used PPIs for more than 1 year found no significant differences between the patients' levels of Vitamin B12 [13]. Another study comparing elderly subjects who have been on PPI for more than 3 years with their non-PPI users found no difference in vitamin B12 levels as well [14].

One can still obtain the desired outcomes for such associations through the use of a Laboratory Information System (LIS) and avoid the expense of repeating tests [15]. Moreover, for older adults, MMA/creatinine urine testing is another non-invasive and cost-effective method for screening vitamin B12 levels [16]. Consequently, Vitamin B12 deficiency is a serious health issue caused by the suppression of ileal membrane transport and intrinsic factor activity, and if misdiagnosed can lead to SCD which is a rare and dangerous complication. Therefore, evaluating its potential risk factors is of concern [17].

In order to gain a deeper understanding, we conducted a cohort analysis to assess the prevalence & association of Vitamin B12 deficiency in patients on long-term use of PPIs.

## 2 Materials and methods

This single-center cohort study was conducted at the Department of Internal Medicine, KRL hospital in Islamabad, Pakistan from May 2021 to May 2022. An estimated sample size of 1225 participants was calculated using a Rao soft calculator keeping a 95% confidence interval & 5% margin of error. Non-probability consecutive sampling technique was used.

### 2.1 Eligibility criteria

Patients aged 18–80 years of both genders, on regular PPI therapy for >1 year were included in our study. Subjects with acquired causes of B_12_ deficiency, such as those with a history of atrophic gastritis, pernicious anemia, gastric bypass, gastrointestinal infection, ileal resection, Crohn's disease, tapeworm infection, alcohol consumption, dietary restrictions (such as vegetarians and vegans), or those on medications such as histamine H_2_ blockers and metformin were excluded.

### 2.2 Ethical approval and consent

Ethical approval was obtained from the Institutional Review Board (IRB) of the KRL hospital, Islamabad, Pakistan before the collection of data. The aim of the study was explained in detail and Consent was taken from every patient prior to data collection. Patients' clinical data were then extracted from patient records using a standardized proforma.

Our study is fully compliant with the STROCSS 2021 guidelines [18]. A complete STROCSS 2021 checklist has been provided as a supplementary file. Our study has been registered on Research Registry with the following UIN: researchregistry8101 [19]. Our study is in accordance with the Declaration of Helsinki.

### 2.3 Data extraction

Sociodemographic and medical details such as age, gender, type of proton pump inhibitor (PPI) prescribed, dosage and duration of its use, baseline Vit. B_12_ levels and after treatment with PPI were extracted.

Vitamin B_12_ levels were analyzed using the Cobas e411 analyzer. Subjects with a B_12_ deficient state were administered an intramuscular injection of cyanocobalamin on alternative days for 2 weeks, followed by oral therapy thrice daily for four to eight weeks.

### 2.4 Analysis

Data was analyzed using SPSS by IBM version 26. Quantitative data were presented as frequencies and percentages. A Chi-square test was applied to see the different associations of B_12_ deficiency. The Odds Ratio was calculated to find the risk of B_12_ deficiency in patients with long-term use of PPI. T-Test was applied to evaluate the impact of initial and final levels of vitamin B12.

## 3 Results

Among the total participants, more than half of the men (55.10%) had low levels of vitamin B12. A p-value of 0.00 was calculated for Vit B12 deficiency in the 18-40-year-old age group as well as in the 41-80-year-old group. similarly, a p-value of 0.00 was also found for the 41-80-year-old age group, as shown in Table 1.

**Table 1:** Patient demographics in association with vit B12 levels checked on initial visit.

Gender	Vit B12 levels on Initial visit		P -value
	10–100	101–200	
Male	675	0	0.00
Female	53	497	
**Age**			
18–40 years	648	0	0.00
41–80 years	80	497	

Vit B12 levels were observed to be significantly lower in Omeprazole patients (54.12%) than in Pantoprazole patients (45.87%). A vitamin B12 deficiency is 0.5 times more likely in patients taking PPIs (OR = 0.560, 95% CI = 0.444–0.707), as shown in Table 2.

**Table 2:** Vit B12 levels in patients on PPI Use.

PPI taken by patients	Vit B12 levels on Initial Visit		P -value	Odds Ratio	95% Confidence Interval
	10–100	101–200			
Omeprazole	352	311	0.00	0.560	0.444–0.707
Pantoprazole	376	186			

561 patients (45.8%) were on PPI usage for more than 2.6 years, 664 patients (54.2%) had been on PPI use for 1–2.5 years in our study, as shown in Table 3.

**Table 3:** Disease duration of patients taking Regular PPI.

Disease Duration	Frequency	Percent
1–2.5 years	664	54.2
2.6 = 5 years	561	45.8

Vit B12 deficiency is more likely to occur 10.3 times in men taking Regular PPI (OR = 10.377, 95% CI = 8.03–13.404). If a patient is between the ages of 18 and 40, they are seven times more likely to have vitamin B12 insufficiency (OR = 7.213, 95% CI = 5.885–8.839), as shown in Table 4.

**Table 4:** Risk estimate of vit B12 on initial visit.

Association of Vit B12 levels on Initial visit	Odds Ratio	95% Confidence Interval
Gender	10.377	8.03–13.404
Age	7.213	5.885–8.839

There were 599 men (88.74%) who stated that their levels were risen between 450 and 625 ng/ml, while 76 men (11.26%) claimed that their levels were raised between 625 and 900 ng/ml 550 females (44.89%) said their Vit B12 levels were between 625 and 900 ng/ml. Six hundred and forty-eight patients were found to have adequate amounts of vitamin B12, of which 599 (92.43%) had levels in the 450–625 ng/ml range and 76 (7.57%) had levels in the 626–900 ng/ml range. Nearly half of the 577 (47.10) individuals who had their vitamin B12 levels above normal were between the ages of 41 and 80, which had a significant p-value, as shown in Table 5.

**Table 5:** Gender & Age association with Vit B12 Levels Post Treatment.

Gender	Vit B12 levels Post Treatment		P -value
	450–625	626–900	
Male	599	76	0.00
Female	0	550	
**Age**			
18–40 years	599	49	0.00
41–80 years	0	577	

Using paired-samples t-tests, researchers were able to determine the effect that initial and final B12 levels had on visiting patients. Patients' B12 levels rose significantly as a result of the study. There is a substantial difference between the early and final levels of B12, with the latter having a mean of 749.3 ± 116.1. The 95% confidence interval for the mean rise in test values was 663.84 ± 124.94. (−670.845 to −656.837), as shown in Table 6.

**Table 6:** Impact of initial & final levels of vitamin B12.

Vit B12 Levels	Mean ± SD	N	Mean diff ±SD	95% Confidence Interval	P-value
**Initial Visit**	85.99 ± 57.46	1225	−663.841 ± 124.94	−670.845 to −656.837	0.00
**Post Treatment**	749.83 ± 116.11	1225			

## 4 Discussion

Our study indicates Using PPIs for 12 months reduced vitamin B12 levels in the body, which was more pronounced among PPI users than those who did not use PPIs. Long-term PPI medication may have an impact on vitamin B12 status.

Vitamin B12 insufficiency may be linked to PPIs, although there is still a lot of debate regarding how to monitor this relationship in recent years. Long-term usage of PPIs may raise the risk of vitamin B12 insufficiency because of vitamin B12 malabsorption and bacterial overgrowth in the digestive tract [20].

In healthy volunteers on short-term omeprazole therapy for two weeks, Marcuard et al. [21] were the first to demonstrate dose-dependent reduction of cyanocobalamin absorption. However, Termanini et al. [22] were the first to report an increased risk for vitamin B12 deficiency due to long-term use of PPIs in a prospective study on patients with Zollinger-Ellison syndrome, similar to our results showing 10.3 times the risk between long-term PPI usage and vitamin B12 insufficiency.

There have been conflicting studies on the absolute safety of PPI in recent years, particularly in patients who have been using it for a long time. Our analysis included 664 patients (54.2%) who had been using PPI for between one and two and a half and two and a half years, as shown in another study [23].

Vit B12 deficiency cannot be treated with the routinely prescribed PPI doses in those who eat a healthy diet, according to research. For those over the age of 40 and those with long-term nutritional issues, there is a two to fourfold increased risk of vitamin B12 deficiency [24], which is similar to our data showing that those between the ages of 18 and 40 are seven times more likely to have vitamin B12 insufficiency. Leiden University Medical Centre study shows Participants who had been taking PPIs for six years or more did not differ from participants who had been using PPIs for three to five years, but our study showed that 664 patients (54.2%) had been on PPI use for more than one to two years were diagnosed with Vit B12 deficiency [14].

A study done at Mercy Medical Center states that reduction in B12 status occurs in older persons who take long-term PPIs but not long-term H2 blockers; supplementation with the RDA amount of B12 does not prevent this decline. Vitamin B12 shortage is frequent in the elderly, and it looks prudent to test B12 status occasionally while on chronic PPI usage [25], although patients between the ages of 18 and 40 are seven times more likely to have vitamin B12 insufficiency than those over the age of 70.

Over half of patients at University Hospital Aachen were female, with 67% of those over 60 and 68% of Caucasians over the age of 18 being female. Long-term PPI use was linked to an increased incidence of vitamin B12 deficiency (odds ratio: 1.7; 95% CI:1.6–1.7) [26]. Whereas among the 675 male participants in our study, only 550 (44.9%) had Vitamin B12 levels below normal. The Prishtina University in Kosovo enrolled a total of 250 adult volunteers, 200 of whom were PPI users and 50 were in the control group. They found significant changes in serum ferritin and vitamin B12 levels among the PPIs group and particular PPIs subgroups between the two-time points [20], which is consistent with our findings that patients' B12 levels climbed significantly as a result of the study. Early and final B12 levels differ significantly, with the latter having a mean value of 749.3 ± 116.1. Test values rose by an average of 663.84 ± 124.94%. From (−670.845) to (−656.837).

### 4.1 Limitations

This cohort is a single-centered study which may lower the variety of cases that could be presented. On the other hand, according to the authors, being a single-centered article has increased the focus of the researchers and has enhanced the accuracy of the study to multiple folds. Secondly, the data collection time span was about a year which led to the enrolment of over 1200 participants, this may also increase the authenticity of the analyses. Thirdly, during the time, when the association between PPIs and VIT B12 deficiency is not clear, this mass research may be of help in reaching the conclusion about this association.

## 5 Conclusion

According to our findings, long-term usage of PPIs is linked to an increased risk of vitamin B12 insufficiency specifically in men falling under the ages of 18 and 40. Using PPIs at the lowest effective dose for the shortest time possible is critical to minimizing side effects. These side effects have the potential to have a therapeutic impact, although their causal link is still under investigation.

## Ethical approval

Ethical Review Committee of KRL Hospital Islamabad has thoroughly reviewed the synopsis of titled “Association of Vitamin B12 Deficiency with Long-Term PPIs Use: A Cohort Study” reference no KRL–HI–ERC/May/0056, Dated: May 01, 2021 which will be conducted in KRL Hospital Islamabad.

## Funding

Not applicable.

## Author contribution

Contributor ship Statement:

1. The main concept was determined by Hassan Mumtaz
2. Data is interpreted by Syeda Tahira Waheed, Hassan Mumtaz
3. Writing of the manuscript is done by Syed Hasan Ali, Kashmala Dahar
4. Manuscript editing is done by Abdul Ahad Syed, Bushra Ghafoor, Hassan Mumtaz
5. Critical Review is done by Hina Saghir and Mariam Tariq

## Registration of research studies

1. Name of the registry: Research Registry
2. Unique Identifying number or registration ID: researchregistry8101
3. Hyperlink to your specific registration (must be publicly accessible and will be checked): Browse the Registry - Research Registry

## Guarantor

Hassan Mumtaz and Hina Saghir.

## Consent

Consent was taken from every patient prior to data collection. Patients' clinical data were then extracted from patient records using a standardized proforma.

## Provenance and peer review

Not Commissioned, externally peer-reviewed.

## Future implications

This study will create awareness to the medical practitioners to discontinue long term prescription of PPI which will in return improve patient care.

## Declaration of competing interest

Nil.
